# Resolved Influenza A Virus Infection Has Extended Effects on Lung Homeostasis and Attenuates Allergic Airway Inflammation in a Mouse Model

**DOI:** 10.3390/microorganisms8121878

**Published:** 2020-11-27

**Authors:** Qingyu Wu, Ilka Jorde, Olivia Kershaw, Andreas Jeron, Dunja Bruder, Jens Schreiber, Sabine Stegemann-Koniszewski

**Affiliations:** 1 Experimental Pneumology, Department of Pneumology, Health Campus Immunology, Infectiology and Inflammation, Otto-von-Guericke University Magdeburg, 39120 Magdeburg, Germany; Qingyu.Wu@st.ovgu.de (Q.W.); Ilka.Jorde@med.ovgu.de (I.J.); Jens.Schreiber@med.ovgu.de (J.S.); 2 Institute of Veterinary Pathology, Department of Veterinary Medicine, Freie Universität Berlin, 14163 Berlin, Germany; Olivia.Kershaw@fu-berlin.de; 3 Infection Immunology Group, Institute of Medical Microbiology, Infection Control and Prevention, Health Campus Immunology, Infectiology and Inflammation, Otto-von-Guericke University Magdeburg, 39120 Magdeburg, Germany; Andreas.Jeron@med.ovgu.de (A.J.); dunja.bruder@med.ovgu.de (D.B.); 4 Immune Regulation Group, Helmholtz Centre for Infection Research, 38124 Braunschweig, Germany

**Keywords:** influenza A virus, allergic asthma, allergic airway inflammation, respiratory immune regulation, pro-inflammatory cytokines, macrophages

## Abstract

Allergic airway inflammation (AAI) involves T helper cell type 2 (Th2) and pro-inflammatory responses to aeroallergens and many predisposing factors remain elusive. Influenza A virus (IAV) is a major human pathogen that causes acute respiratory infections and induces specific immune responses essential for viral clearance and resolution of the infection. Beyond acute infection, IAV has been shown to persistently affect lung homeostasis and respiratory immunity. Here we asked how resolved IAV infection affects subsequently induced AAI. Mice infected with a sublethal dose of IAV were sensitized and challenged in an ovalbumin mediated mouse model for AAI after resolution of the acute viral infection. Histological changes, respiratory leukocytes, cytokines and airway hyperreactivity were analyzed in resolved IAV infection alone and in AAI with and without previous IAV infection. More than five weeks after infection, we detected persistent pneumonia with increased activated CD4^+^ and CD8^+^ lymphocytes as well as dendritic cells and MHCII expressing macrophages in the lung. Resolved IAV infection significantly affected subsequently induced AAI on different levels including morphological changes, respiratory leukocytes and lymphocytes as well as the pro-inflammatory cytokine responses, which was clearly diminished. We conclude that IAV has exceptional persisting effects on respiratory immunity with substantial consequences for subsequently induced AAI.

## 1. Introduction

Due to constant interactions of the lungs and airways with the outside environment, there is a strong need for the regulation of respiratory immune responses [1]. Immunological homeostasis can be disturbed through acute triggers such as infections and during chronic respiratory inflammation. Throughout the last years, increasing attention has been paid to the cross-talk between such immunological triggers, e.g., in the context of viral/bacterial co-infections, of the respiratory microbiome and of exacerbations of chronic inflammatory respiratory diseases [1,2,3].

Influenza A virus (IAV) is a major human pathogen with a zoonotic reservoir and high pandemic potential. In humans, IAV causes respiratory infections that can range from mild symptoms to life-threatening viral pneumonia [4]. IAV induces effective host responses including the generation of CD4^+^ and CD8^+^ T cell responses as well as B cell-dependent antibody responses [5,6,7]. At times, the host response can be excessive leading to a so-called cytokine storm [8,9]. Next to IAV mediated cell death, also viral clearance by depletion of infected host cells leads to substantial collateral damage [5,9]. Regulatory, anti-inflammatory and repair mechanisms, in which the respiratory epithelium is a central site, are in place to protect from such immune-pathology and to promote recovery [5,7,9,10]. Especially in acute IAV infection, enhanced susceptibility to secondary bacterial infections is a major complication, which at least in part is attributed to the antiviral immune and especially the cytokine response [11,12,13,14]. In secondary bacterial infections following IAV infection, inadequate inflammatory responses are frequently observed and can be both dampened as well as overshooting [13,15]. We and others have shown that IAV infection affects the lung and the respiratory immune system for extended periods of time following clearance of the virus and resolution of the infection [12,16,17,18,19,20]. Robust clinical data on long-term effects of IAV infection on respiratory health and especially respiratory immunity are however lacking. Nevertheless, lasting effects on patient lung function have been reported up to one year following IAV infections [21,22,23,24].

In mouse models, IAV-associated effects on respiratory immune responses are not restricted to secondary infections but have likewise been reported for non-infectious inflammatory conditions such as allergic airway inflammation (AAI) [25,26,27]. The underlying mechanisms however remain largely elusive. Allergic asthma in humans is a chronic respiratory disease affecting up to 300 million people world-wide [28]. Generally, asthma is characterized by reversible airway obstruction, airway hyperreactivity as well as respiratory inflammation and considerably affects the quality of life of patients [29]. Different endotypes have been defined and one discrimination is that between non-allergic (intrinsic) and allergic (extrinsic) asthma. Allergic asthma is frequent and is typically associated with T helper cell type 2 (Th2) responses. These include the production of Th2 cytokines such as interleukin (IL)-4, IL-5 and IL-13, eosinophilic inflammation and allergic responses to aeroallergens involving allergen-specific IgE antibodies, mast cells and basophils [30]. In allergic asthma, major open questions relate to predisposing factors as well as to the drivers of different inflammatory phenotypes [31,32]. In childhood, not only infections with rhinovirus and respiratory syncytial virus are associated with the subsequent development of allergic asthma [7,33]. Furthermore, the number of respiratory episodes has been shown to play a role independently of the infectious agent [34]. This is less clear for infection-triggered adult-onset asthma [35].

In previous studies in a mouse model, following sublethal infection with IAV A/PR/8/34 (H1N1) we observed effective viral clearance by day 10 post infection but persistent bronchointerstitial pneumonia as late as three weeks and increased susceptibility to secondary pneumococcal infection for up to two weeks [12,36]. Here, we now tested the hypothesis that IAV infection would affect lung immune homeostasis for even longer and that this would have implications for subsequently induced AAI. We analyzed mice on day 39 post sublethal IAV infection and observed strong persistent changes to the respiratory immune cell composition. At the same time, AAI induced after the resolution of IAV infection was significantly altered with respect to histological changes, the recruitment of effector cells as well as the pro-inflammatory cytokine response. These results underline the significance of IAV as a potential threat to respiratory health that acts long after the acute infection and has implications beyond susceptibility to secondary bacterial infections.

## 2. Materials and Methods

### 2.1. Mice

All experiments were performed in 7–8 week old female specific-pathogen free C57Bl/6 mice. Mice were obtained from Janvier (Saint-Berthevin, France), housed in individually ventilated cages in groups of 3–5 and were fed food and water ad libitum. All experiments were ethically reviewed and approved by the responsible authorities (Landesverwaltungsamt Sachsen-Anhalt, file number 203.6.3-42502-2-1495).

### 2.2. Influenza A Virus Infection

IAV A/PR/8/34 (H1N1) was propagated in Madin–Darby canine kidney cells as previously described [36]. Mice were anaesthetized through intraperitoneal (i.p.) injection of ketamine-xylazine and intranasally (i.n.) infected with 0.31 TCID_50_ (50% tissue culture infectious dose) IAV A/PR/8/34 (H1N1) diluted in 25 µL PBS as described elsewhere [12]. Control mice were administered 25 µL PBS i.n.

### 2.3. Induction of Allergic Airway Inflammation

Two weeks after IAV infection or control treatment, mice were sensitized i.p. with 10 µg ovalbumin (OVA, grade V, Sigma-Aldrich, St. Louis, MO, USA) in PBS containing 1 mg aluminum hydroxide (alum; Imject^TM^ Alum Adjuvant, Thermo Fisher) in weekly intervals (day 14, 21, 28). Control mice were mock-sensitized i.p. with alum only. One week after the last sensitization, on three consecutive days (day 35, 36, 37) all mice were i.n. challenged with 100 µg OVA (grade III, Sigma-Aldrich) in 30 µL PBS under light isoflurane anesthesia. Forty-eight hours after the last challenge (day 39), mice were sacrificed and bronchoalveolar lavage (BAL), serum and lungs were harvested for further analyses.

### 2.4. Serum

Blood was collected post mortem, incubated 20 min at 37 °C and 5 min at 4 °C and centrifuged 10 min at 4 °C (1500× *g*). Serum was aliquoted and stored at −80 °C until further analysis.

### 2.5. Isolation of Leukocytes

Lungs were flushed once through the trachea with 1 mL ice-cold, sterile PBS to obtain BAL. The supernatant (360× *g*, 10 min, 4 °C) was cleared from debris (10,000× *g*, 5 min, 4 °C) and stored at −80 °C. Erythrocyte lysis through osmotic shock was performed on the cell pellet and cells were subjected to flow cytometric analyses. Lavaged lungs were perfused with 10 mL ice-cold PBS through the heart, excised and minced on ice. Tissue degradation was performed by enzymatic digestion (45 min at 37 °C) in Iscove’s modified Dulbecco’s medium containing 0.2 mg/mL Collagenase D (Sigma-Aldrich), 0.01 mg/mL DNase (Sigma-Aldrich) and 5% fetal calf serum. EDTA was added to a final concentration of 5 mM and suspensions were filtered (70 µm). Erythrocyte lysis by osmotic shock was performed and leukocytes were enriched using Percoll (1.041 g/mL) (GE Healthcare Life Sciences).

### 2.6. Flow Cytometry

Flow cytometry was performed as described [37]. Briefly, cells were incubated with anti-CD16/CD36 (2.4G2) to block Fc-receptors and were stained with fixable live/dead stain (BioLegend). Antibody staining for B220 (RAE6B2), CD3 (17A2), CD4 (RM4-5 or GK1.5), CD11b (M1/70), CD11c (N418), CD49b (HMα2), CD69 (H1.2F3), CD117 (2B8), FcεRIα (MAR-1), Ly6G (1A8), MHCII (M5/114.15.2), NK1.1 (PK136) and Siglec-F (E50-2440, ThermoFisher, Waltham, MA, USA) was performed in different combinations (see below). Antibodies were obtained from BioLegend unless otherwise indicated. Data were acquired using an Attune NxT instrument (ThermoFisher) and were analyzed using the FlowJo software (Tree Star). Cells were gated following singlet-gating and dead cell exclusion. Single stainings were performed for all fluorochromes for compensation using UltraComp eBeads (ThermoFisher) and fluorescence-minus-one stainings were performed for gating. For the calculation of absolute cell numbers from the relative frequencies, 50,000 fluorescent beads (Precision Count Beads, BioLegend) were added to each sample.

Leukocytes from BAL were gated for CD11c^+^, CD11b^+^/CD11c^−^ and CD11b^−^/CD11c^−^ cells (Figure S1). CD11c^+^ cells were gated for macrophages (CD11c^+^/Siglec-F^+^) and Siglec-F^−^ cells, from which dendritic cells (DC) (CD11c^+^/Siglec-F^−^/MHCII^+^) were gated using MHCII as a marker. Neutrophils were gated as CD11b^+^/CD11c^−^/Ly6G^+^ cells and eosinophils were gated as CD11b^+^/CD11c^−^/Ly6G^−^/Siglec-F^+^ cells. CD4^+^ T cells were gated as CD11b^−/^CD11c^−^/CD4^+^ and CD8^+^ T cells as CD11b^−^/CD11c^−^/CD8^+^ cells.

For the analysis of lung macrophages, DC and lymphocytes, the cells were initially gated for CD11c^+^ and CD11c^-^ cells (Figure S2). CD11c^+^ cells were further analyzed for Siglec-F and MHCII. Macrophages were gated as CD11c^+^/Siglec-F^+^ cells. The MHCII expression level of CD11c^+^/Siglec-F^+^ macrophages was analyzed as the median fluorescence intensity (MFI) of the MHCII signal of the CD11c^+^/Siglec-F^+^ macrophage population of a sample normalized to the mean MHCII MFI of the control group (PBS/alum only/OVA) of that experiment. DC were gated as CD11c^+^/Siglec-F^−^/MHCII^+^ cells. CD11c^−^ cells were further gated by using the B220 and MHCII markers. B cells were gated as CD11c^−^/B220^+^/MHCII^+^ cells and CD11c^−^/B220^−^/MHCII^−^ cells were gated for CD4^+^ T cells (CD11c^−^/B220^−^/MHCII^−^/CD4^+^) and CD8^+^ T cells (CD11c^-^/B220^−^/MHCII^−^/CD8^+^). Th2 cells were gated as ST2^+^ CD4^+^ T cells. CD4^+^ T cells, CD8^+^ T cells and Th2 cells were analyzed for CD69 expression.

For the analysis of lung eosinophils, neutrophils and mast cells, CD3^−^/NK1.1^−^/B220^−^/CD11b^+^ cells were gated for neutrophils (CD3^−^/NK1.1^−^/B220^−^/CD11b^+^/Ly6G^+^/Siglec-F^−^) and eosinophils (CD3^−^/NK1.1^−^/B220^−^/CD11b^+^/Ly6G^−^/Siglec-F^+^) in a separate staining (Figure S3). Mast cells (FcεRIα ^+^/CD117^+^/CD49^−^) were gated from singlets without prior dead cell exclusion.

### 2.7. ELISA

Serum OVA-specific IgE was detected by enzyme-linked immunoassay (ELISA) according to the manufacturer’s recommendations (LEGEND MAX^TM^ Mouse OVA Specific IgE ELISA Kit). The absorbance was measured at 450 nm (Tecan Infinite^®^ M Plex Photometer) and data were analyzed using Microsoft Excel.

### 2.8. Quantification of Cytokines in BAL

Cytokines were quantified in undiluted BAL using a 13-plex cytometric bead array according to the manufacturer’s instructions (LEGENDplex^TM^ Th cytokine panel, BioLegend). IL-2 (2.22 pg/mL), IL-4 (1.34 pg/mL), IL-5 (4.07 pg/mL), IL-6 (0.69 pg/mL), IL-9 (1.22 pg/mL), IL-10 (6.65 pg/mL), IL-13 (1.70 pg/mL), IL-17A (2.14 pg/mL), IL-17F (1.85 pg/mL), IL-21 (1.72 pg/mL), IL-22 (2.15 pg/mL), IFN-γ (1.39 pg/mL) and TNF-α (2.09 pg/mL) were analyzed (detection limits).

### 2.9. Assessment of Airway Hyperreactivity

Assessment of airway hyperreactivity was performed as described [37]. Anesthetized and tracheotomized mice were mechanically ventilated (Buxco FinePointe R/C, DSI^TM^ USA) and after 5 min of acclimation, 10 μL PBS containing increasing concentrations of methacholine (0, 6.25, 12.5, 25, 50 mg/mL) were nebulized into the breathing air (delivery duration 20 s). Resistance and compliance were assessed over a three minute response time for each concentration. Before nebulization of the next higher methacholine concentration there was a one minute recovery time. Using the continuously measured lung pressure and airflow values, resistance and compliance were calculated based on the single compartment lung model. Data were analyzed using the FinePointe software.

### 2.10. Histopathological Analysis

Histopathological analyses were performed as described [37]. Whole lungs were fixed in 4% formalin and embedded in paraffin. Sections (5 μm) were dewaxed and stained with hematoxylin and eosin and a veterinary pathologist certified by the European College of Veterinary Pathologists performed blinded histological evaluations. The proportion (in %) of the affected tissue was assessed and lungs were scored (1 = mild, 2 = moderate; 3 = high) for perivascular lymphocytic infiltrates, interstitial lymphocytic infiltrates, alveolar lymphocytes, interstitial eosinophils, alveolar eosinophils, alveolar neutrophils, bronchial epithelial hyperplasia and type II pneumocyte hyperplasia. Following PAS (periodic-acid Schiff) reaction, accumulation of mucus and goblet cell hyperplasia in the medium sized and large bronchi were assessed.

### 2.11. Statistical Analysis

Data for all experimental groups were tested for normality using the Shapiro–Wilk normality test. In the case of Gaussian distribution for all groups in a comparison, an unpaired two-sided t-test or one-way ANOVA and Bonferroni post-hoc test was performed. In the case of non-Gaussian distribution in at least one of the groups in a comparison, an unpaired two-sided Mann–Whitney test or a Kruskal–Wallis test with Dunn’s post-hoc test was performed. To assess the effects of IAV infection alone in our experimental setup, direct comparison of IAV infected, mock-sensitized mice (IAV/alum only/OVA) and PBS-treated, mock-sensitized mice (PBS/alum only/OVA) was performed (t-test or Mann–Whitney test). To test for the induction of AAI, comparison of PBS-treated, sensitized mice (PBS/OVA-alum/OVA) and IAV infected, sensitized mice (IAV/OVA-alum/OVA) to the PBS-treated, mock-sensitized control group (PBS/alum only/OVA) was performed (one-way ANOVA or Kruskal–Wallis test). To assess the effects of resolved IAV infection on AAI, direct comparison between PBS-treated, sensitized mice (PBS/OVA-alum/OVA) and IAV infected, sensitized mice (IAV/OVA-alum/OVA) was performed (*t*-test or Mann–Whitney test). *p* ≤ 0.05 was considered indicative of statistical significance (* *p* < 0.05, ** *p* < 0.01, *** *p* < 0.005, **** *p* < 0.0001). All statistical analyses were performed using the Graph Pad Prism software version 8 (Graph Pad Software). The groups’ medians were used to calculate the fold-changes indicated in the text.

## 3. Results

### 3.1. Influenza A Virus Infection Results in Persisting Changes in the Pulmonary Immune Cell Composition

**Figure 1:**
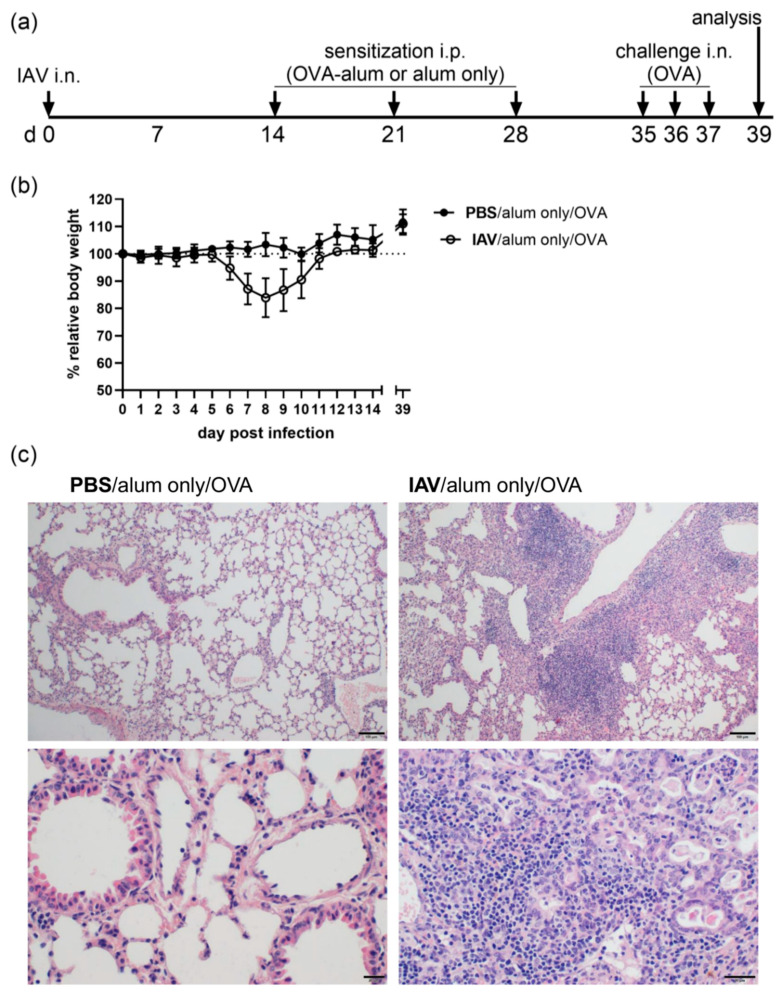
IAV (influenza A virus) infection leads to persistent histological changes in the lung. (**a**) Mice were infected i.n. with a sublethal dose of IAV or treated with PBS on day 0, were mock-sensitized (alum only i.p.; days 14, 21, 28) and treated i.n. with OVA (days 35, 36, 37). (**b**) Body weight was monitored daily following the infection. Relative body weights are shown as the mean ± SD of *n* = 19 controls (PBS/alum only/OVA) and *n* = 23 IAV infected mice (IAV/alum only/OVA) for days 0 to 14 and day 39 compiled from five experiments. (**c**) On day 39 post infection, mice were sacrificed and lung tissue was histologically analyzed. Representative images are shown for one out of 4 controls (PBS/alum only/OVA) and one out of 5 infected mice (IAV/alum only/OVA) analyzed. The upper images were prepared at 100× magnification (scale bar 100 µm), the lower images at 400× magnification (scale bar 20 µm).

To assess persisting changes of IAV infection to the lung and their effects on AAI as a secondary non-infectious trigger, mice were first infected with a sublethal dose of IAV A/PR/8/34 (H1N1). Following recovery, mice were sensitized through i.p. injection of OVA together with alum or mock-sensitized with alum only (days 14, 21 and 28). Following sensitization or mock-sensitization, all mice were challenged i.n. with OVA (days 35, 36 and 37) and analyzed two days later (day 39) (Figure 1a). As previously described, IAV infection led to transient weight loss from which mice recovered by day 14 post infection (Figure 1b). In previous studies employing the same virus and sublethal infection model, we have demonstrated that mice have cleared the virus by this time-point [12,36]. By day 39 post IAV infection, mice had further gained body weight while at the same time histological analyses revealed persistent infection-associated changes affecting around 25% of the lung (Figure 1b,c and Figure S4). While there were mild lymphocytic infiltrations also in the uninfected controls (in ≤10% of the lung, Figure S4a), moderate to severe bronchointerstitial pneumonia was detected following resolution of IAV infection. Here, pneumonia presented as subacute to chronically active. Changes were multi-focal to confluent and were detected predominantly closely to the hilum. Specifically, we observed moderate to severe, mainly lymphocytic to lymphoplasmacytic cuffs as well as moderate to severe interstitial infiltrates. In the alveoli of the affected areas there was mild accumulation of neutrophils, mild accumulation of moderately activated macrophages and there were few lymphocytes. Furthermore, there was mild hyperplasia of bronchial epithelial cells and type II pneumocytes (Figure S4).

**Figure 2:**
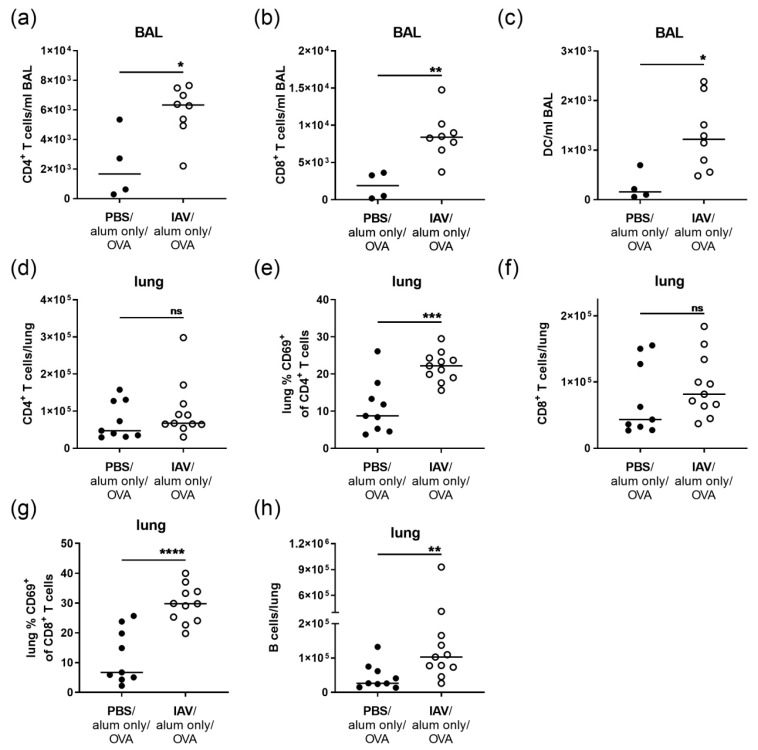
Resolved IAV infection leads to elevated T cell numbers in the BAL and to persistent T cell activation as well as accumulation of B cells in the lung. Mice were infected i.n. with a sublethal dose of IAV or treated with PBS on day 0, were mock-sensitized (alum only i.p.; days 14, 21, 28) and treated i.n. with OVA (days 35, 36, 37). On day 39 post infection, CD4^+^ T cell (**a**), CD8^+^ T cell (**b**) and DC (**c**) numbers in the BAL (bronchoalveolar lavage) were flow cytometrically analyzed. In the lung, CD4^+^ T cell numbers (**d**), CD69-expression of CD4^+^ T cells (**e**), CD8^+^ T cell numbers (**f**), CD69-expression of CD8^+^ T cells (**g**) and B cell numbers (**h**) were analyzed. Data are shown for individual mice together with the group median. BAL data are compiled from at least two independent experiments and lung data are compiled from three independent experiments. * *p* < 0.05, ** *p* < 0.01, *** *p* < 0.005, **** *p* < 0.001, ns = not significant.

Prompted by the histological analyses, we performed flow cytometric analyses of leukocytes isolated from the BAL and lung tissue on day 39 post IAV infection (Figure 2). Here, we detected a slight increase but no significant change in the total numbers of BAL leukocytes between the controls and infected mice (Figure S5). At the same time there was a strong and significant increase in BAL CD4^+^ T cells (3.8-fold), CD8^+^ T cells (4.4-fold) and DC (7.9 fold) following IAV infection (Figure 2a–c). In the lung, the total numbers of leukocytes were likewise not significantly changed (Figure S5). While lung CD4^+^ T cell numbers were not significantly elevated following IAV infection, they revealed a strong and significant increase in the expression of the activation marker CD69 (2.5-fold increase) (Figure 2d,e). Similar results were obtained for lung CD8^+^ T cell numbers and CD69-expression (4.4-fold increased) (Figure 2f,g). Moreover, resolved IAV infection resulted in significantly increased B cell numbers in the lungs by day 39 post infection (3.9-fold increase) (Figure 2h).

**Figure 3:**
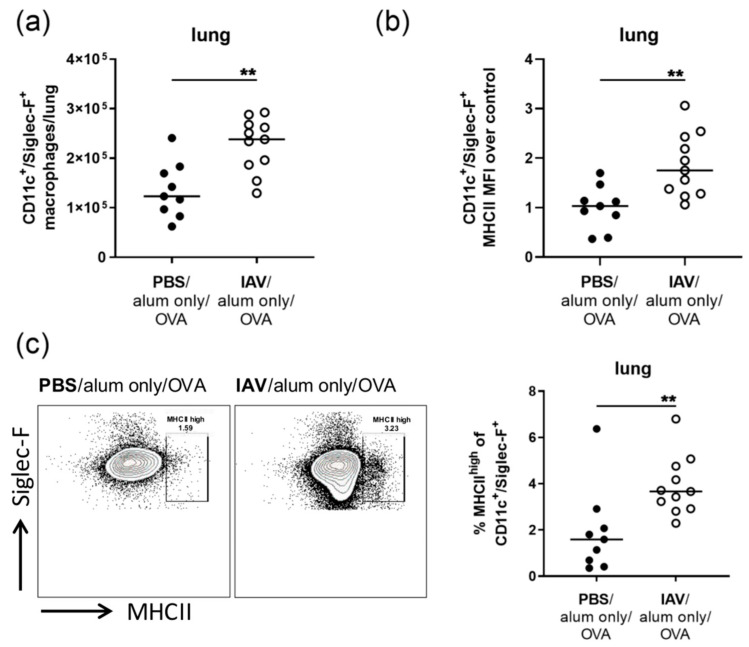
Resolved IAV infection leads to the accumulation of CD11c^+^/Siglec-F^+^ macrophages with increased MHCII expression the lung. Mice were infected i.n. with a sublethal dose of IAV or treated with PBS on day 0, were mock-sensitized (alum only i.p.; days 14, 21, 28) and treated i.n. with OVA (days 35, 36, 37). On day 39 post infection, CD11c^+^/Siglec-F^+^ macrophage numbers (**a**), CD11c^+^/Siglec-F^+^ macrophage MHCII expression (MFI, median fluorescence intensity) (**b**) and the frequency of MHCII^high^ CD11c^+^/Siglec-F^+^ macrophages in the CD11c^+^/Siglec-F^+^ macrophage population (**c**) were flow cytometrically analyzed. Data are shown for individual mice together with the group median. Representative flow cytometry plots show MHCII expression of the gated CD11c^+^/Siglec-F^+^ macrophage population of an uninfected and an infected mouse. Data are compiled from three independent experiments. ** *p* < 0.01.

Furthermore, there were significant changes in the lung CD11c^+^/Siglec-F^+^ macrophage population, with significantly increased numbers of CD11c^+^/Siglec-F^+^ macrophages following IAV infection (1.9-fold increase) (Figure 3a). Their median MHCII expression was significantly increased as compared to uninfected controls (1.7-fold) (Figure 3b) and a population of CD11c^+^/Siglec-F^+^ macrophages with high MHCII expression was observed (Figure 3c).

Taken together, we observed substantial alterations to the respiratory immune cell composition long after IAV infection both in histological as well as in flow cytometric analyses. These changes mainly comprised an increase in lymphocytes, DC and CD11c^+^/Siglec-F^+^ macrophages as well as increased T cell activation and macrophage MHCII expression.

### 3.2. Resolved IAV Infection Affects the Histological Presentation of AAI

**Figure 4:**
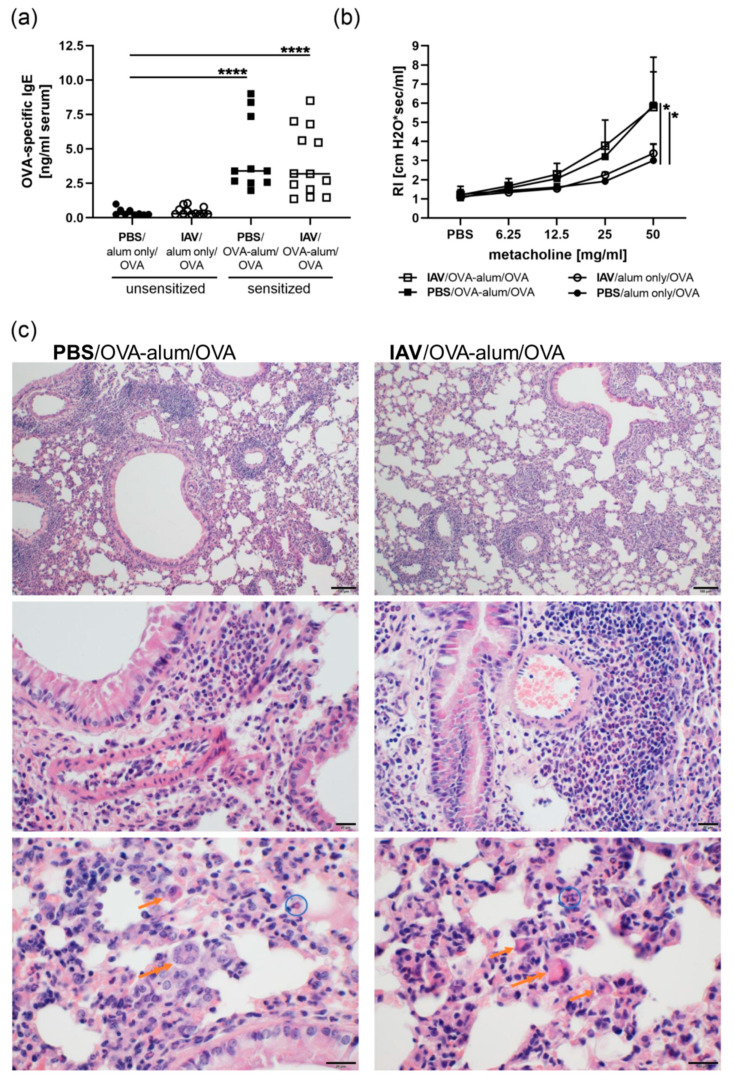
Resolved IAV infection does not affect IgE production and airway hyperreactivity but affects histological changes in AAI. Mice were infected i.n. with a sublethal dose of IAV or treated with PBS on day 0, were sensitized against OVA i.p or mock-sensitized with alum only i.p. on days 14, 21 and 28 and all mice were challenged i.n. with OVA on days 35, 36 and 37. On day 39 post infection, OVA-specific IgE levels in the serum (**a**), airway hyperreactivity in terms of lung resistance (RI) in response to metacholine (**b**) and histological changes in hematoxylin and eosin stained lung tissue (**c**) were analyzed. In (**a**), data are shown for individual mice compiled from three independent experiments together with the group median. In (**b**) the mean + SD of *n* = 4 control mice (PBS/alum only/OVA) and *n* ≥ 5 IAV infected only (IAV/alum only/OVA), sensitized only (PBS/OVA-alum/OVA) or IAV infected and sensitized mice (IAV/OVA-alum/OVA) compiled from two independent experiments are shown. *p*-values in (**b**) refer to RI following administration of 50 mg/mL metacholine. In (**c**), representative images for one out of 5 analyzed mice/group are shown. The upper images were prepared at 100× magnification (scale bar 100 µm), the images of the middle panel at 400× magnification (scale bar 20 µm) and the lower panel at 600× magnification (scale bar 20 µm). Orange single arrows point at alveolar histiocytosis, double arrows at multinucleated macrophages and blue circles at eosinophils. * *p* < 0.05, **** *p* < 0.001.

In an OVA mediated murine model for AAI we analyzed the consequences of previous IAV infection and the associated respiratory changes on the phenotype of AAI (Figure 1a). Mice sensitized and challenged with OVA produced significant levels of OVA-specific IgE antibodies detectable in the serum and IgE production was not altered by previous IAV infection (Figure 4a). Airway hyperreactivity to unspecific triggers such as metacholine is another hallmark feature of AAI. As for the IgE response, the sensitization and respiratory challenge with OVA induced significant hyperreactivity to metacholine, which also was not affected by previous IAV infection (Figure 4b). Importantly, while airway reactivity was enhanced as expected in AAI, this was not the case following IAV infection alone (Figure 4b). Histologically, AAI presented as moderate to severe, chronic-active, particularly eosinophilic bronchointerstitial pneumonia (Figure 4c and Figure S4). Histological changes were located throughout the parenchyma and were characterized by moderate, mainly lymphocytic cuffs and moderate interstitial, often eosinophilic as well as lymphocytic infiltrations. Affected areas of the lung furthermore showed mild accumulation of eosinophils, noticeably activated macrophages (multinucleated giant cells) and lymphocytes in the alveoli. Furthermore, mild bronchial epithelial hyperplasia and type II pneumocyte hyperplasia were observed (Figure S4). Moreover, we observed moderate goblet cell hyperplasia in the medium sized bronchi and severe hyperplasia of goblet cells in the large bronchi, while accumulation of mucus was scarce (Figure S6). Altogether, AAI induced following IAV infection presented similarly as mild to severe, chronic-active particularly eosinophilic bronchointerstitial pneumonia (Figure 4c and Figure S4). Changes in AAI induced following IAV infection as opposed to AAI alone were the detection of moderate as opposed to mild alveolar eosinophils and a severe as opposed to mild accumulation of activated macrophages (giant cells) (Figure S4). Furthermore, bronchial epithelial and pneumocyte type II hyperplasia was severe in AAI following IAV infection as opposed to mild in AAI alone or following IAV infection alone (Figure S4). Accumulation of mucus as well as goblet cell hyperplasia in the large bronchi was unchanged between AAI alone and AAI induced following IAV infection, while goblet cell hyperplasia in the medium-sized bronchi was mainly mild in AAI induced following IAV infection as opposed to moderate in AAI alone (Figure S6).

Taken together, AAI was likewise induced without changes in IgE production or airway hyperreactivity following IAV infection. Histological analyses suggested that previous IAV infection had effects mainly on AAI-associated epithelial hyperplasia and the accumulation of activated histiocytes.

**Figure 5:**
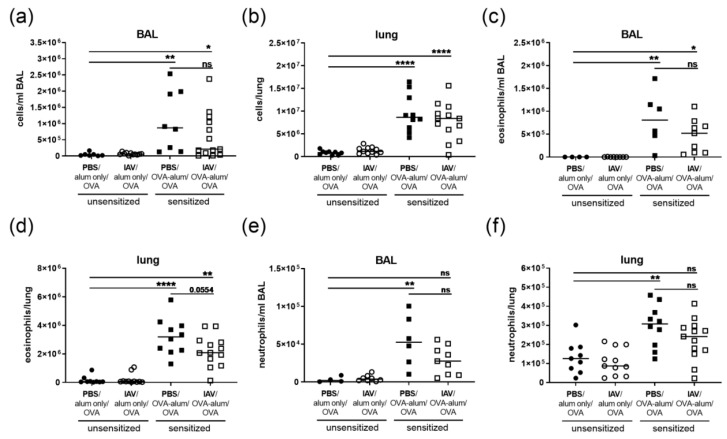
Resolved IAV infection does not affect respiratory tract total cell and eosinophil numbers but reduces respiratory neutrophils in AAI. Mice were infected i.n. with a sublethal dose of IAV or treated with PBS on day 0, were sensitized against OVA i.p or mock-sensitized with alum only i.p. on days 14, 21 and 28 and all mice were challenged i.n. with OVA on days 35, 36 and 37. On day 39 post infection, total leukocyte numbers in the BAL (**a**) and lungs (**b**), eosinophil numbers in the BAL (**c**) and lungs (**d**) and neutrophil numbers in the BAL (**e**) and lungs (**f**) were flow cytometrically analyzed. Data are shown for individual mice together with the group median. BAL data are compiled from two to three independent experiments and lung data are compiled from three independent experiments. * *p* < 0.05, ** *p* < 0.01, **** *p* < 0.001, ns = not significant.

**Figure 6:**
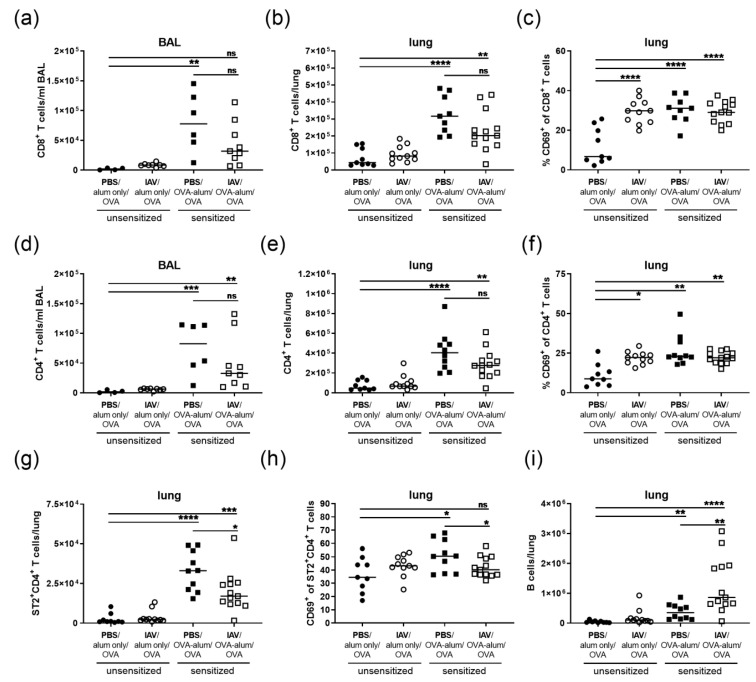
Resolved IAV infection significantly affects lung Th2 cell numbers, Th2 cell activation and B cell numbers in AAI. Mice were infected i.n. with a sublethal dose of IAV or treated with PBS on day 0, were sensitized against OVA i.p or mock-sensitized with alum only i.p. on days 14, 21 and 28 and all mice were challenged i.n. with OVA on days 35, 36 and 37. On day 39 post infection, CD8^+^ T cell numbers in the BAL (**a**) and lungs (**b**), the frequency of CD69-expressing CD8^+^ T cells in the lungs (**c**), CD4^+^ T cell numbers in the BAL (**d**) and lungs (**e**), the frequency of CD69-expressing CD4^+^ T cells in the lungs (**f**), Th2 cell (ST2^+^CD4^+^ T cells) numbers in the lungs (**g**), the frequency of CD69-expressing Th2 cells in the lung (**h**) and lung B cell numbers (**i**) were flow cytometrically analyzed. Data are shown for individual mice together with the group median. BAL data are compiled from two independent experiments and lung data are compiled from three independent experiments. * *p* < 0.05, ** *p* < 0.01, *** *p* < 0.005, **** *p* < 0.001, ns = not significant.

Based on the extended changes in the lung immune cell composition detected following IAV infection alone and the histopathological alterations we observed in AAI, we analyzed leukocytes in the respiratory tract in AAI with and without previous IAV infection (Figure 5 and Figure 6). In the BAL and the lung, we detected significantly increased total cell numbers in AAI alone as well as in AAI preceded by IAV infection as compared to uninfected, mock-sensitized controls (Figure 5a,b). There were no significant differences in BAL or lung total cell numbers between AAI alone and AAI preceded by IAV infection. Furthermore, as compared to the controls there was a significant increase in the numbers of eosinophils in the BAL and lungs in AAI alone and in AAI preceded by IAV infection. Median eosinophil numbers were lower if AAI was preceded by IAV infection (BAL 1.6-fold decrease; lung 1.5-fold decrease as compared to AAI alone), which however did not reach statistical significance (lung *p* = 0.0554) (Figure 5c,d). Neutrophil numbers in the BAL and lung were significantly increased in AAI alone as compared to the control group. If AAI was preceded by IAV infection, neutrophil numbers were lower as compared to AAI alone (BAL 1.9-fold decrease; lung 1.3-fold decrease) and at the same time, as compared to the uninfected, mock-sensitized control there was no significant increase in neutrophil numbers in the BAL or the lung if AAI was preceded by IAV infection (Figure 5e,f). Numbers of lung mast cells, key effector cells in AAI, were likewise significantly increased both in AAI alone as well as in AAI preceded by IAV infection (Figure S7). The same was true for CD11c^+^/Siglec-F^+^ macrophages, which were increased in AAI with and without previous IAV infection to a similar extent as following IAV infection alone (Figure S7). Furthermore, the median MHCII expression of CD11c^+^/Siglec-F^+^ macrophages was likewise increased in IAV infection alone, AAI alone and AAI preceded by IAV infection (Figure S7). Moreover, the population of MHCII^high^ CD11c^+^/Siglec-F^+^ macrophages was increased in AAI without effects of preceding IAV infection (Figure S7). Taken together, BAL and lung total cell numbers and more specifically eosinophils, mast cells and CD11c^+^/Siglec-F^+^ macrophages in AAI were not affected by preceding IAV infection. At the same time the recruitment of neutrophils to the respiratory tract was decreased in AAI preceded by IAV infection.

As T and B lymphocytes are important effector cells in AAI and as we had detected clear extended effects of IAV infection on respiratory lymphocytes (Figure 2), we furthermore analyzed the effects of resolved IAV infection on the respiratory lymphocyte compartment in AAI (Figure 6). In the BAL, AAI alone led to a strong and significant increase in CD8^+^ T cell numbers. These were lower (2.4-fold reduction as compared to AAI alone) and not significantly elevated as compared to the control if AAI was preceded by IAV infection (Figure 6a). In the lung, CD8^+^ T cell numbers were significantly increased in AAI with and without previous IAV infection (Figure 6b). We furthermore analyzed CD69 expression of CD8^+^ T cells in the lung, which was strongly increased in AAI independently of whether it was preceded by IAV infection or not (Figure 6c). Of note, CD69-expression of CD8^+^ T cells was equally high also following resolution of IAV infection alone. CD4^+^ T cell numbers were significantly increased in the BAL and lungs in AAI alone as well as in AAI induced following IAV infection as compared to the uninfected, mock-sensitized controls (Figure 6d,e). It is noteworthy that in the BAL and lungs both CD8^+^ (BAL 2.4-fold; lung 1.6-fold reduction as compared to AAI alone) and CD4^+^ T cell numbers (BAL 2.5-fold; lung 1.5-fold reduction as compared to AAI alone) were lower if AAI was preceded by IAV infection, even though this reduction did not reach statistical significance (Figure 6a,b,d,e). CD69-expression of CD4^+^ T cells was clearly and similarly elevated following resolution of IAV infection alone and in AAI, independent of whether it was preceded by IAV infection or not (Figure 6f). While the effects of previous IAV infection on AAI were not significant regarding CD4^+^ and CD8^+^ T cell numbers, we detected clear effects on lung Th2 cells and B cells (Figure 6g–i). The induction of AAI alone led to a clear and significant increase in lung Th2 cells. This was also the case if AAI was preceded by IAV infection, however with significantly lower Th2 cell numbers (1.9-fold decrease as compared to AAI alone) (Figure 6g). CD69-expression in lung Th2 cells was significantly elevated in AAI alone but not in AAI preceded by IAV infection as compared to the controls (Figure 6h). At the same time, Th2 cell CD69-expression was significantly reduced in AAI preceded by IAV infection as compared to AAI alone (1.25-fold decrease). Lung B cell numbers were significantly increased in AAI alone and in AAI induced following IAV infection as compared to the unsensitized control group (Figure 6i), with significantly increased B cell numbers in AAI preceded by IAV infection (2.5-fold increase as compared to AAI alone).

Taken together, resolved IAV infection significantly reduced the recruitment and activation of Th2 cells in the lung and significantly increased lung B cell numbers in AAI.

**Figure 7:**
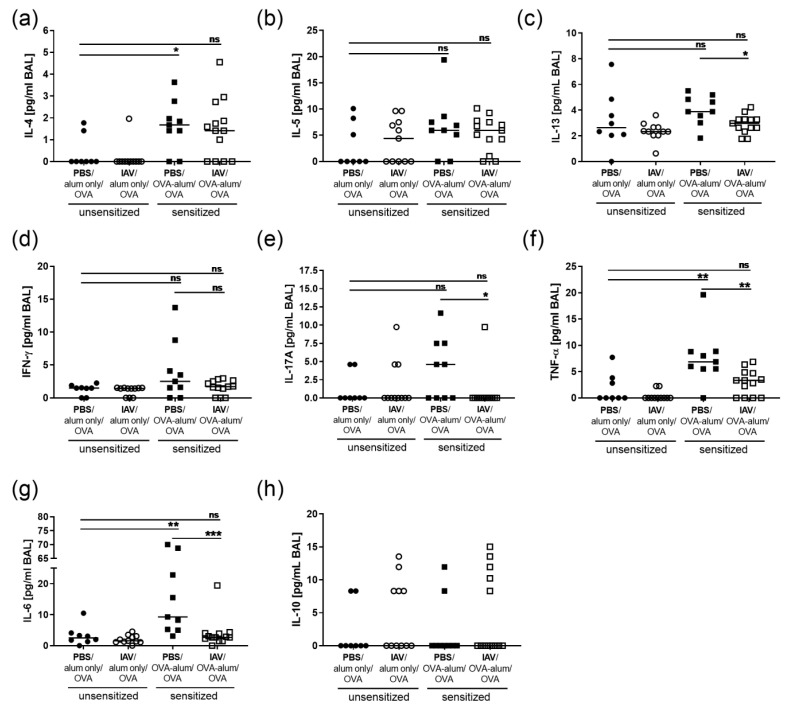
Resolved IAV infection significantly inhibits the pro-inflammatory cytokine response in AAI. Mice were infected i.n. with a sublethal dose of IAV or treated with PBS on day 0, were sensitized against OVA i.p or mock-sensitized with alum only i.p. on days 14, 21 and 28 and all mice were challenged i.n. with OVA on days 35, 36 and 37. On day 39 post infection, BAL levels of IL-4 (**a**), IL-5 (**b**), IL-13 (**c**), IFN-γ (**d**), IL-17A (**e**), TNF-α (**f**), IL-6 (**g**) and IL-10 (**h**) were analyzed. Data are shown for individual mice together with the group median. BAL samples were collected in three independent experiments. * *p* < 0.05, ** *p* < 0.01, *** *p* < 0.005, ns = not significant.

Cellular responses in respiratory infection and inflammation are associated with the release of cytokines. For further insight into inflammation in AAI with and without previous IAV infection, we assessed respiratory cytokine levels. Of note, following resolution of IAV infection none of the analyzed cytokines were significantly elevated (Figure 7). AAI alone led to a significant elevation of the Th2 cytokine IL-4 in the BAL (Figure 7a). Respiratory IL4 was also detected in AAI preceded by IAV infection, there was however no significant increase as compared to the mock-sensitized control group. IL-5 was not significantly elevated in AAI alone or if preceded by IAV infection (Figure 7b). The median IL-13 level was marginally, however not significantly, increased in AAI alone as compared to the uninfected, mock-sensitized control group (Figure 7c). At the same time, IL-13 was significantly lower in AAI preceded by IAV infection (1.3-fold reduction) as compared to AAI alone (Figure 7c). Furthermore, the Th1 cytokine IFN-γ was sporadically detected in AAI alone but was not significantly elevated in either AAI alone or in AAI preceded by IAV infection as compared to the mock-sensitized control group (Figure 7d). As for IL-13, also the production of IL-17A was generally not significantly elevated in AAI but was significantly reduced in AAI preceded by IAV infection as compared to AAI alone (Figure 7e). In contrast, respiratory production of the pro-inflammatory cytokines TNF-α and IL-6 was clearly and significantly induced in AAI alone (Figure 7f,g). TNF-α was also slightly elevated in AAI preceded by IAV infection but not significantly changed as compared to the mock-sensitized controls and significantly reduced (2.1-fold decrease) as compared to AAI alone (Figure 7f). IL-6 was hardly induced in AAI preceded by IAV infection and at the same time IL-6 levels were clearly and significantly decreased (3.5-fold reduction) as compared to AAI alone (Figure 7g). This dampening of the pro-inflammatory cytokine response in AAI by preceding IAV infection was not associated with an induction of anti-inflammatory IL-10 following resolution of IAV infection alone or in AAI preceded by IAV infection (Figure 7h).

Taken together, resolved IAV infection at different levels affected respiratory cytokine production in AAI, especially through ameliorating the production of the pro-inflammatory cytokines TNF-α and IL-6.

## 4. Discussion

Influenza viruses are major respiratory pathogens that, depending on the circulating viral strains, can infect up to 20% of the human population during the winter months [38]. Even though clinical data of long-term consequences of influenza infections on respiratory health are rare, substantial effects on lung function have been reported even for mild IAV infections [23]. In a combined mouse model for sublethal IAV infection and AAI we show that IAV infection has extended effects on the immunological microenvironment of the lung and that it significantly affects the lymphoid and myeloid cellular composition and the pro-inflammatory cytokine response in subsequent allergic inflammation.

Previous studies in animal models have assessed long-term effects of IAV infection on the lung. Epithelial changes associated with repair mechanisms have been reported up to 200 days post infection with PR8 IAV infection [18], mucous cell metaplasia has been reported up to 21 days post PR8 infection [39] and goblet cell hyperplasia up to 28 days post X31 IAV infection [40]. Furthermore, Keeler and colleagues have recently described extensive chronic changes to the lung long after IAV infection [19]. For at least up to 6 months post WS/33 IAV infection, they specifically observed chronic lung disease that was associated with focal bronchiolization as well as mucus production and that was in part linked to IL-13. Furthermore, a long-term increase in respiratory CXCL-1 mRNA has been described 7 weeks following PR8 IAV infection [41]. Histologically, we have previously observed sustained lymphocytic infiltrations up to 21 days post PR8 IAV infection [12]. Here, we have now extended the time-frame post infection to day 39 and have performed detailed analyses of histological changes, lung leukocytes, respiratory cytokines and airway hyperreactivity. In histology, we also observed epithelial hyperplasia but mucus production and goblet cell hyperplasia were absent. Nevertheless, also at this late time point we histologically observed substantial infiltrations pointing at bronchointerstitial pneumonia. These observations were in line with the flow cytometric detection of significantly increased CD4^+^ and CD8^+^ T cell numbers in the BAL more than five weeks post infection. Furthermore, we observed lasting and strong CD69-expression in lung CD4^+^ and CD8^+^ T cells. These changes were independent of any significant levels of Th1, Th2 or pro-inflammatory cytokines in the respiratory tract. We observed significantly increased numbers of DC in the BAL on day 39 post IAV infection, which was well in line with a report describing increased numbers of DC in the lungs on day 30 post HKx31 IAV infection [27]. In that study, lung DC analysis following resolved IAV infection revealed increased co-stimulatory molecule surface expression that was, however, as opposed to our findings, linked to enhanced allergic disease and Th1 and Th2 responses. Next to increased DC numbers in the BAL, we observed mild alveolar histiocytosis and clearly increased numbers of CD11c^+^/Siglec-F^+^ macrophages with at the same time significantly increased MHCII expression in the lung on day 39 post infection. Interestingly, the identification of an interstitial macrophage subset with immune-regulatory properties was recently described and observed to expand following IAV infection [42]. Furthermore, likewise increased numbers of CD11c^+^/Siglec-F^+^ macrophages were observed on day 28 post IAV infection (X31) when they conferred protection from secondary *S. pneumoniae* infection through increased production of IL-6 [16]. As in our study, these post-influenza macrophages showed increased MHCII expression. At earlier time points, as opposed to protection, IAV leaves hosts susceptible to severe secondary bacterial pneumonia [12]. Among other mechanisms, this has been attributed to desensitization of alveolar macrophages to bacterial toll-like receptor ligands that lasts for months after the primary viral infection and was associated with an attenuated recruitment of neutrophils and pro-inflammatory cytokine response [17], as we have observed in AAI induced following resolved IAV infection. It has previously been suggested that the extended effects of IAV infection are heterogeneous and, e.g., depend on the viral strain and dose as well as the severity of the viral infection [18,19]. Nevertheless, altogether a number of studies including ours emphasize a fundamental potential of IAV infection to shape the respiratory microenvironment far beyond the acute infection. Clearly, future studies will be needed to identify common immunological mechanisms and the collection of far more patient data will be essential to possibly translate these finding into clinical applications or prophylactic measures such as an assessment of post-flu risk parameters.

It is widely accepted that infectious and inflammatory events can lead to persistent changes with substantial effects on subsequent immune responses [7,43]. Moreover, a concept of innate trained immunity reaching beyond classical immunological memory of the adaptive immune system has formed [44,45]. Due to the early recognition of the viral/bacterial synergism and its devastating consequences for the host, secondary bacterial infections have been a major focus regarding sequelae of IAV infections. Indeed, multiple mechanisms of IAV-associated regulation of subsequent antibacterial immune responses have been described [13]. In a broader context, it is conceivable that such regulation also affects non-infectious inflammatory conditions such as AAI and previous studies in different mouse models have indeed described significant effects of IAV infection [25,26,27,40,46,47]. In line with the heterogeneous IAV mediated effects described and discussed above, also the consequences for subsequent AAI are heterogeneous and include enhanced sensitization [25,26], aggravated allergic inflammation [27,46] and suppression of AAI [40,47]. Clinical data are mainly restricted to altered anti-IAV responses in asthmatics and IAV mediated acute exacerbations of existing allergic asthma, while the role of influenza viruses in asthma-development is mostly unclear [48,49,50]. Prompted by our previous observation that IAV infection had prolonged effects on the lung that lasted beyond viral clearance [12,36], we were specifically interested in further characterization of these extended effects and how they would affect subsequently induced AAI late after infection. Indeed, we show that AAI in mice sensitized after apparent recovery from IAV infection and challenged 5 weeks after the infection was significantly modulated. While allergen-specific IgE production and airway hyperreactivity were not affected, our major findings were that bronchial epithelial and alveolar type II pneumocyte hyperplasia and the accumulation of activated macrophages and giant cells were strongly amplified by previous IAV infection. On the level of respiratory lymphocytes and cytokines we observed an increase in lung B cell numbers while lung neutrophil recruitment, Th2 cell numbers, Th2 cell CD69-expression and BAL IL-13, IL-17, IL-6 and TNF-α were altogether significantly reduced in AAI preceded by IAV infection. These results point at an attenuation of the Th2 and possibly Th17 response together with a clear suppression of the pro-inflammatory response. This was somewhat unexpected, as especially Th1 and Th2 responses are believed to represent an equilibrium [51]. Others have described suppression of Th2 cell recruitment in AAI following IAV infection that was associated with reduced eosinophil numbers and at the same time an increased Th1 response [40]. Interestingly, also respiratory mucosal vaccination with a whole IAV vaccine prevented Th2 inflammation in AAI through a shift towards a Th1 response [52]. Furthermore, selective TLR7-triggering, which occurs in innate recognition of IAV infection [4], was shown to suppress Th2 responses [53].

The mechanisms underlying IAV mediated suppression or aggravation of AAI and Th2 inflammation in many points remain elusive and the factors decisive for either protection or amplification are unknown. On the one hand, IAV infections have been shown to transiently accumulate key effectors in AAI such as mast cells, group 2 innate lymphoid cells and eosinophils [54,55], which we however did not observe late after IAV infection in our model. While we observed suppression especially of the pro-inflammatory response, without significant effects on IL-5 and eosinophil recruitment, others have observed broader protection from AAI that was mediated by T effector memory cells [47,56]. Although we also observed a lasting increase in respiratory T cells following IAV infection, we believe additional innate mechanisms along the epithelial-macrophage axis at play. We observed an increase in lung macrophages more than five weeks following IAV infection and strong alveolar histiocytosis with an accumulation of giant cells in AAI preceded by IAV infection. At the same time epithelial hyperplasia was strongly increased in AAI preceded by IAV infection as opposed to resolved IAV infection or AAI alone. IAV as well as other respiratory viral infections have been shown to induce trained immunity in respiratory macrophages that protects from secondary bacterial challenge [16,57]. Interestingly, increased MHCII expression as we observed on CD11c^+^/Siglec-F^+^ macrophages in the lung following IAV infection and in AAI, was described for those memory alveolar macrophages induced following IAV infection [57]. It is likely that such trained macrophage immunity also affects allergic mechanisms [45]. Indeed, protection from AAI through replacement of resident alveolar macrophages with regulatory monocytes has been shown for respiratory infection with murid herpesvirus, which however represents a different class of viral pathogen and in which replenishment of lost AM differs from that in IAV infection [58,59]. In our model system, we observed formation of giant cells from activated macrophages that was sporadic in AAI alone but frequent in AAI preceded by IAV infection. There are little reports on such cells in AAI, but very recently granulocyte-macrophage colony stimulating factor (GM-CSF), IL-13 and IL-33 have been shown to mediate their formation in a house dust mite mediated model of AAI [60]. Their function however is unknown. Furthermore, whether their frequent formation in AAI preceded by IAV infection as we describe it here is associated with the modulation of inflammation remains to be elucidated. Cells of the respiratory epithelium strongly react to acute IAV infection [61], regeneration following resolution of the infection is associated with epithelial hyperplasia [62] and there are long-term changes to the respiratory epithelium following IAV infection [18]. Moreover, the respiratory epithelium is broadly involved in asthma pathogenesis [63,64]. Therefore, our finding that bronchial and alveolar hyperplasia was strongly increased in AAI preceded by IAV infection as compared to IAV or AAI alone furthermore suggests the respiratory epithelium to possibly be involved in the observed modulation of AAI by previous IAV infection.

Our study describes prolonged effects of IAV infection on respiratory immunity that have a clear potential to modulate AAI, possibly along a macrophage-epithelial cell axis. We believe that our observations strengthen the emerging concept of trained innate immunity in allergic disorders and further emphasize the need to clarify the role of IAV in shaping inflammation in allergic asthma. Major future challenges will be the experimental and clinical investigation of the exact underlying immunological mechanisms, which will be essential to open novel pathways in the prophylaxis and treatment of allergic asthma.

## Supplementary Materials

The following are available online at https://www.mdpi.com/2076-2607/8/12/1878/s1, Figure S1: Flow cytometry gating strategy for cells isolated from bronchoalveolar lavage (BAL); Figure S2: Flow cytometry gating strategy for the analysis of lung macrophages, DC and lymphocytes; Figure S3: Flow cytometry gating strategy for the analysis of lung eosinophils, neutrophils and mast cells; Figure S4: Histopathological analyses of lung tissue following resolution of IAV infection alone, AAI alone and AAI preceded by IAV infection; Figure S5: Resolved IAV infection does not significantly affect total cell numbers in the bronchoalveolar lavage (BAL) or the lung; Figure S6: Histopathological analyses of mucus production following resolution of IAV infection alone, AAI alone and AAI preceded by IAV infection; Figure S7: Resolved IAV infection does not affect lung mast cell numbers and macrophage numbers or macrophage MHCII expression in AAI.
