# Supplementary material for: Resolved Influenza A Virus Infection Has Extended Effects on Lung Homeostasis and Attenuates Allergic Airway Inflammation in a Mouse Model
Source: Microorganisms. 2020 Nov 27;8(12):1878. doi: 10.3390/microorganisms8121878 (PMC7761027; doi:10.3390/microorganisms8121878)
Supplement: Supplementary file 1 [file microorganisms-08-01878-s001.pdf]

Resolved Influenza A Virus Infection Has Extended Effects on Lung Homeostasis and Attenuates Allergic Airway Inflammation in a Mouse Model

Supplementary Material

(a)

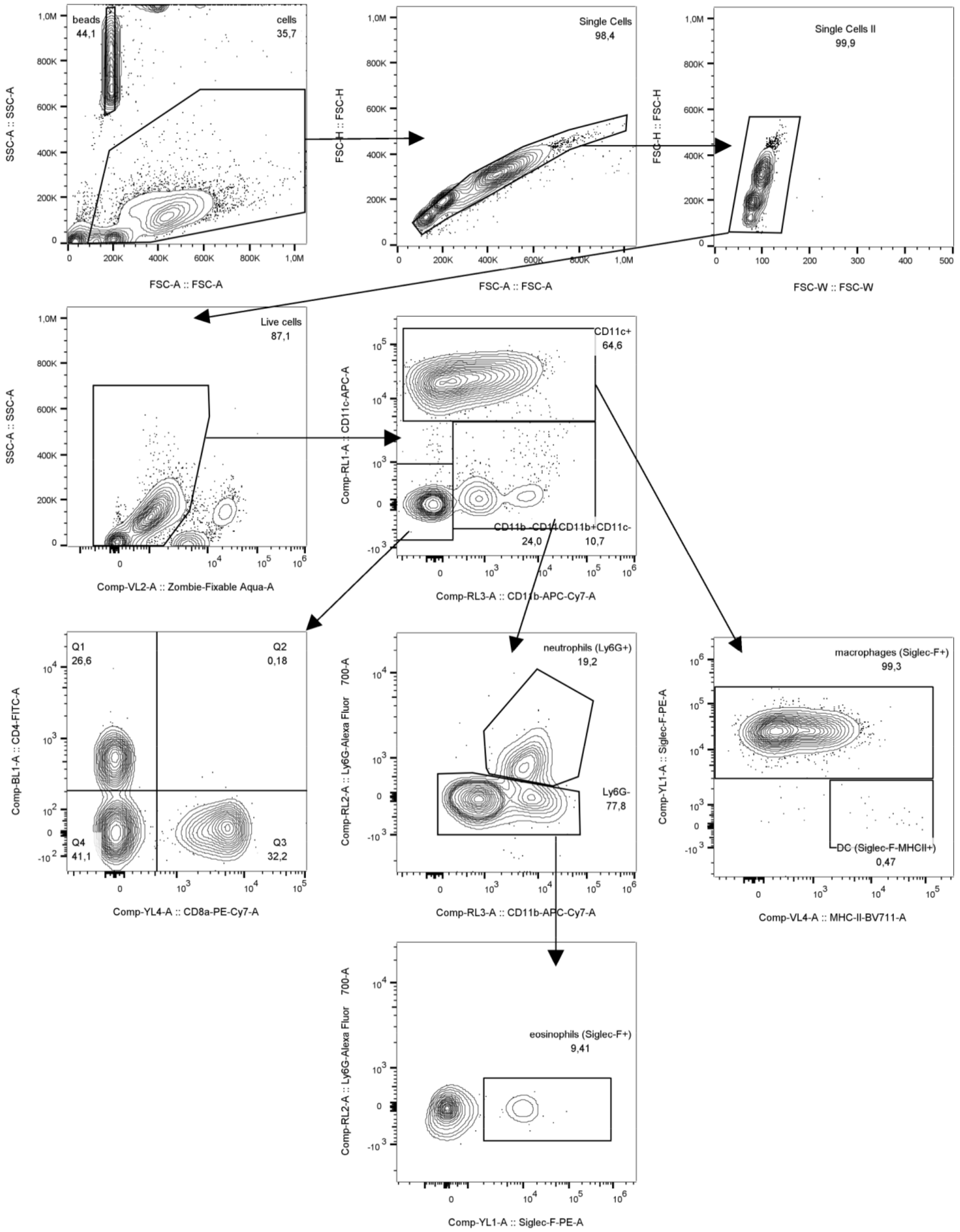
beads
44,1
cells
35,7
SSC-A :: SSC-A
FSC-A :: FSC-A
Single Cells
98,4
FSC-H :: FSC-H
Single Cells II
99,9
FSC-W :: FSC-W
Live cells
87,1
Comp-VL2-A :: Zombie-Fixable Aqua-A
CD11c+
64,6
CD11b−CD11c−
24,0
CD11b+CD11c−
10,7
Comp-RL1-A :: CD11c-APC-A
Comp-RL3-A :: CD11b-APC-Cy7-A
Q1
26,6
Q2
0,18
Q3
32,2
Q4
41,1
Comp-BL1-A :: CD4-FITC-A
Comp-YL4-A :: CD8a-PE-Cy7-A
neutrophils (Ly6G+)
19,2
Ly6G-
77,8
Comp-RL2-A :: Ly6G-Alexa Fluor 700-A
macrophages (Siglec-F+)
99,3
DC (Siglec-F-MHCII+)
0,47
Comp-YL1-A :: Siglec-F-PE-A
Comp-VL4-A :: MHC-II-BV711-A
eosinophils (Siglec-F+)
9,41

(b)

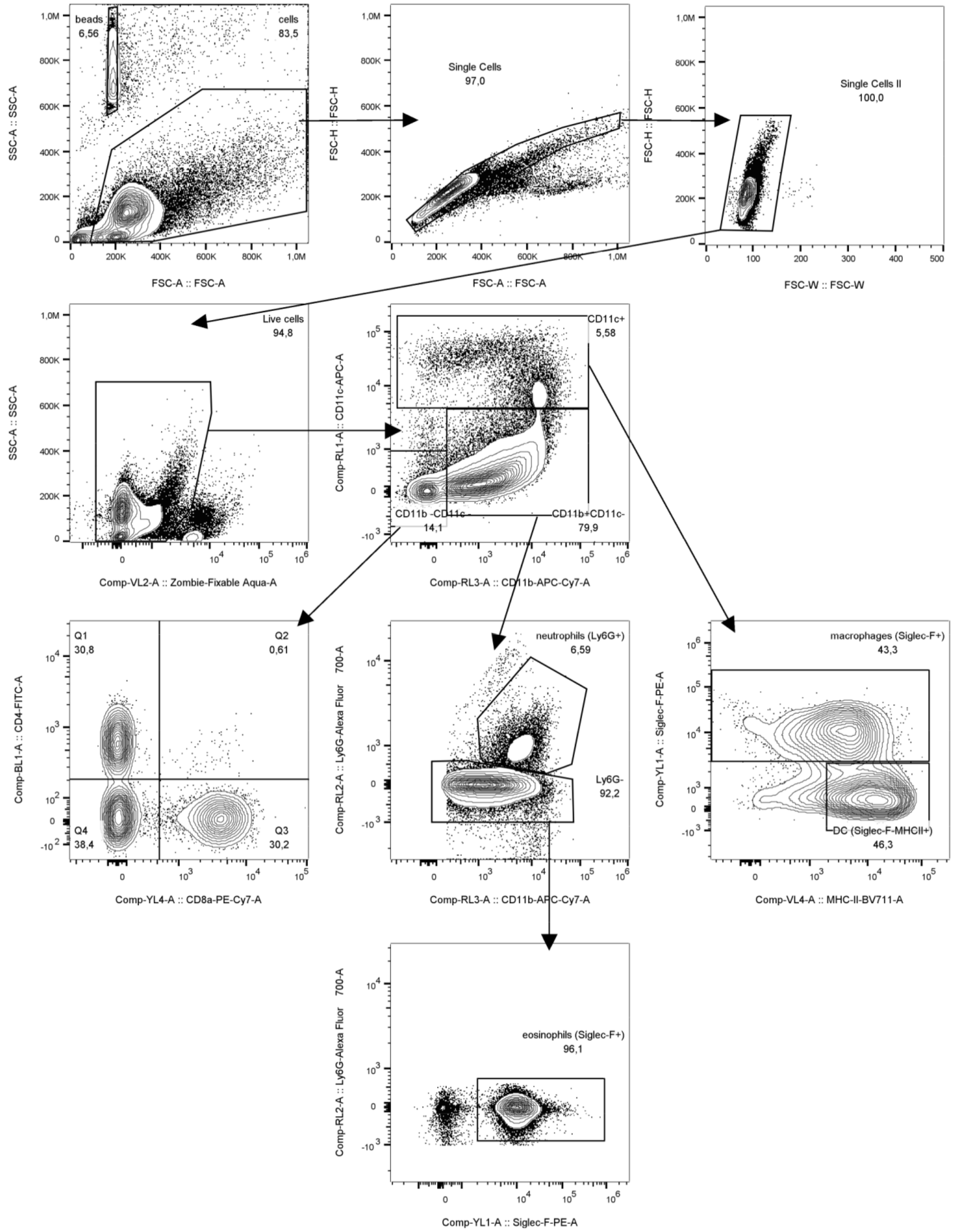

Figure S1. Flow cytometry gating strategy for cells isolated from bronchoalveolar lavage (BAL). Leukocytes from BAL were gated for singlets and live cells. Live cells were gated for CD11c$^+$, CD11b$^+$/CD11c$^-$ and CD11b$^-$/CD11c$^-$ cells. CD11c$^+$ cells were gated for macrophages (CD11c$^+$/Siglec-F$^+$) and Siglec-F$^-$ cells, from which dendritic cells (DC) (CD11c$^+$/Siglec-F$^-$/MHCII$^+$) were gated using MHCII as a marker. Neutrophils were gated as CD11b$^+$/CD11c$^-$/Ly6G$^+$ cells and eosinophils were gated as CD11b$^+$/CD11c$^-$/Ly6G$^-$/Siglec-F$^+$ cells. CD4$^+$ T cells were gated as CD11b$^-$ /CD11c$^-$/CD4$^+$ and

CD8+ T cells as CD11b-/CD11c-/CD8+ cells. Gating is shown for a representative control mouse (PBS/alum only/OVA) **(a)** as well as a representative mouse following the induction of AAI alone (PBS/OVA-alum/OVA) **(b)**. Gate frequencies relate to the respective parent gate.

(a)

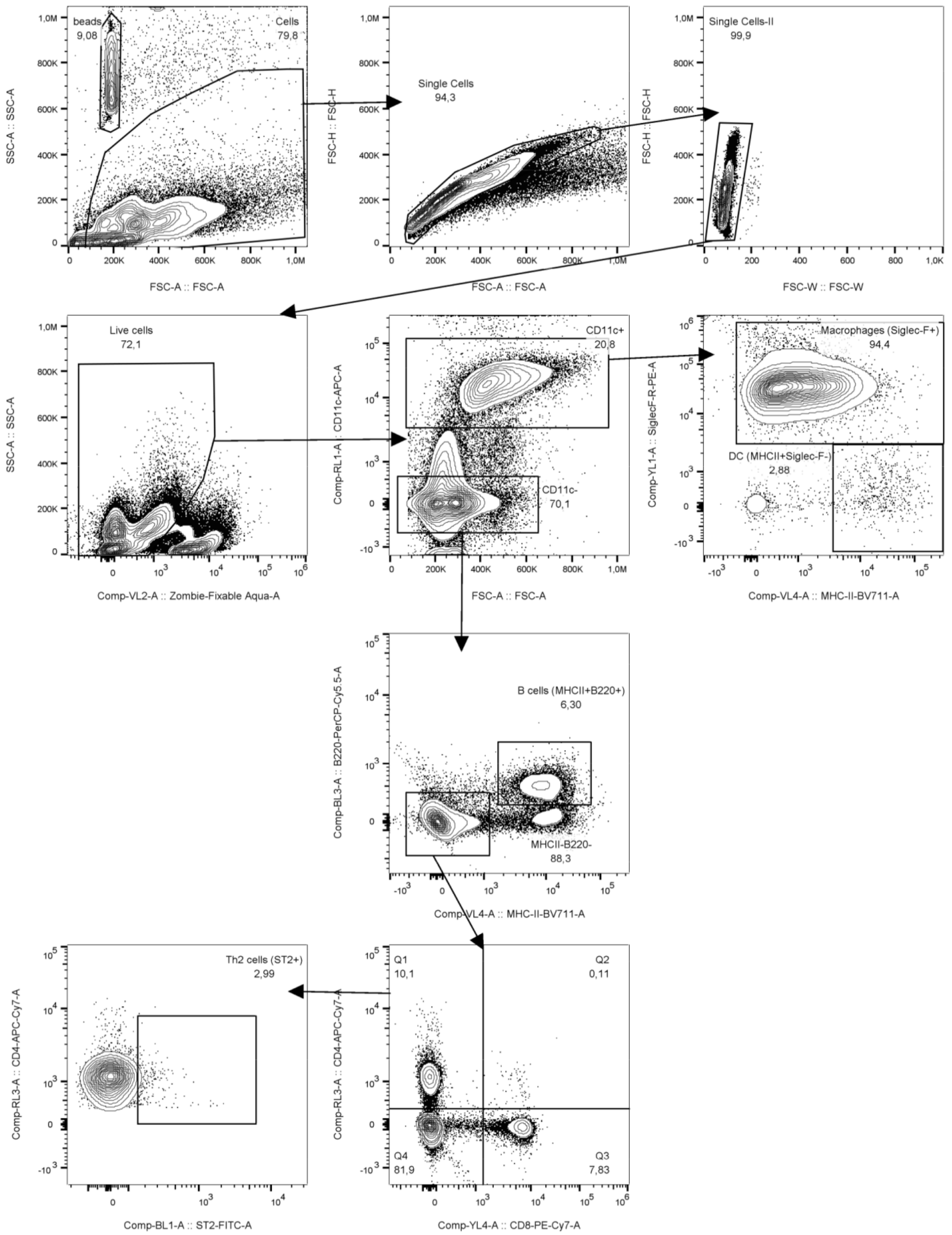

(b)

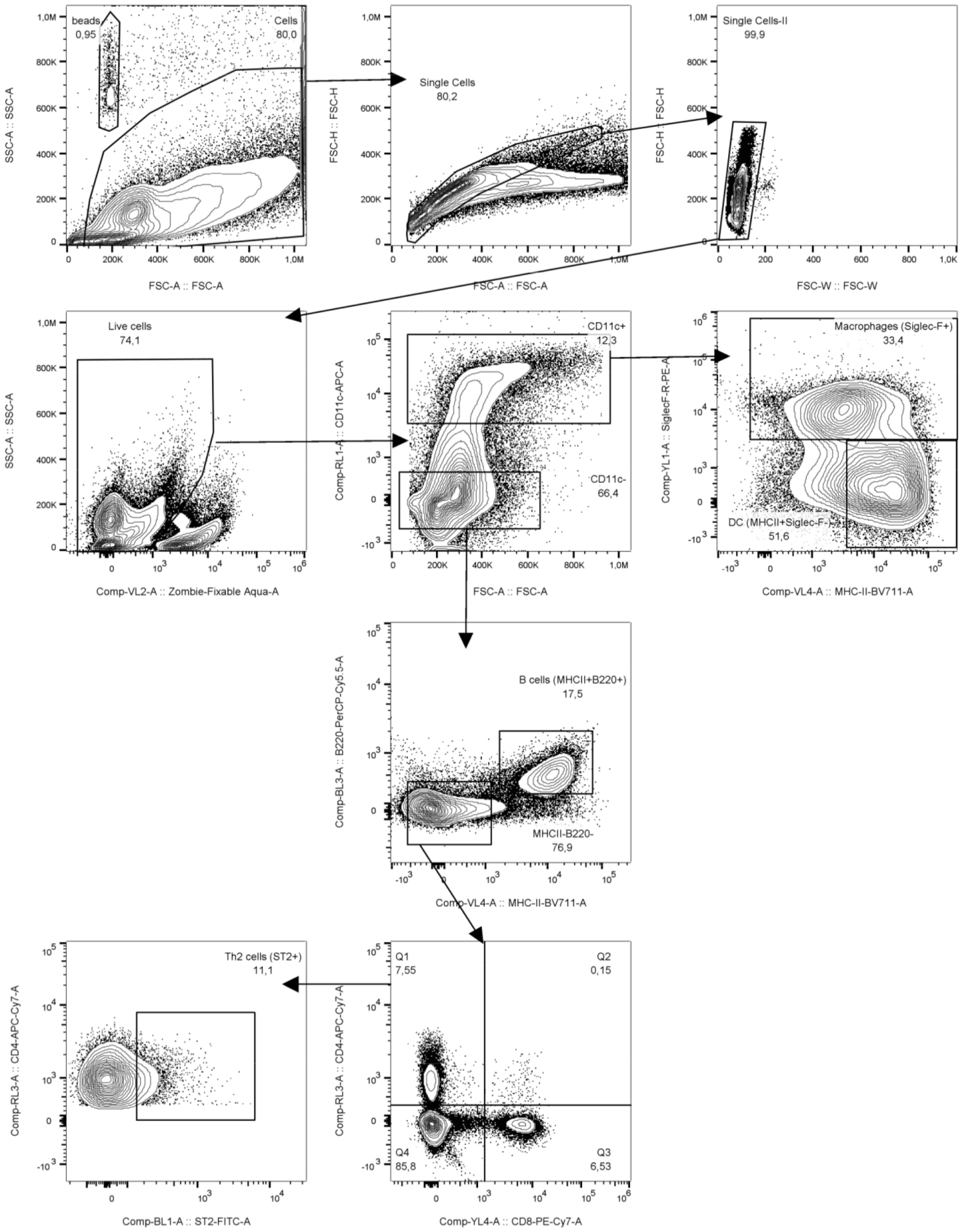

Figure S2. Flow cytometry gating strategy for the analysis of lung macrophages, DC and lymphocytes. Leukocytes from the lung were gated for singlets and live cells. Live cells were gated for $CD11c^+$ and $CD11c^-$ cells. $CD11c^+$ cells were further analyzed for Siglec-F and MHCII. Macrophages were gated as $CD11c^+$/Siglec-F^+ cells. DC were gated as $CD11c^+$/Siglec-F^-/$MHCII^+$ cells. $CD11c^-$ cells were further gated by using the B220 and MHCII markers. B cells were gated as $CD11c^-$/$B220^+$/$MHCII^+$ cells and $CD11c^-$/$B220^-$/$MHCII^-$ cells were gated for $CD4^+$ T cells ($CD11c^-$/$B220^-$/$MHCII^-$/$CD4^+$) and $CD8^+$ T cells ($CD11c^-$/$B220^-$/$MHCII^-$/$CD8^+$). Th2 cells were gated as $ST2^+$ $CD4^+$ T cells. Gating is shown for a

representative control mouse (PBS/alum only/OVA) **(a)** as well as a representative mouse following the induction of AAI alone (PBS/OVA-alum/OVA) **(b)**. Gate frequencies relate to the respective parent gate.

(a)

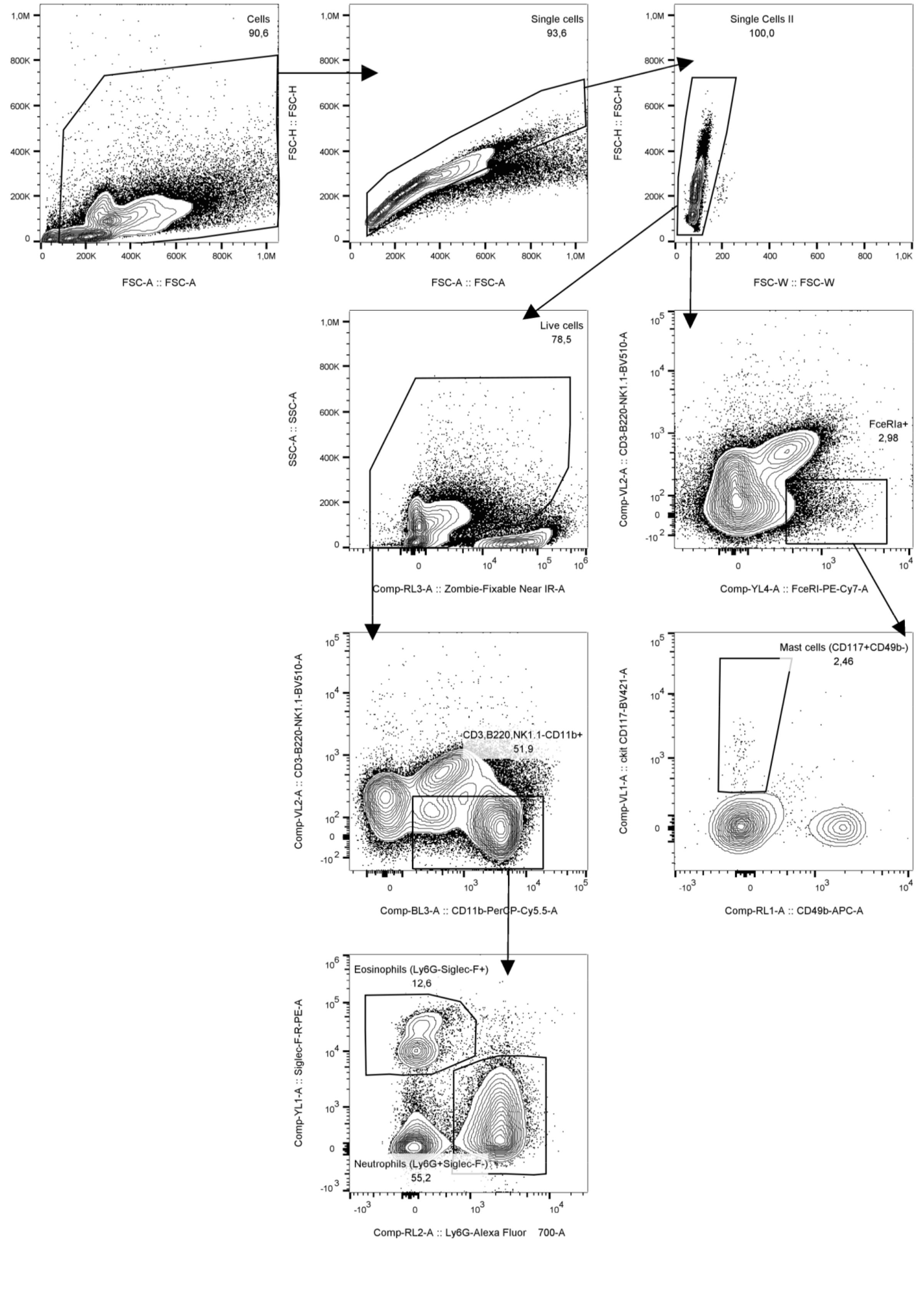

(b)

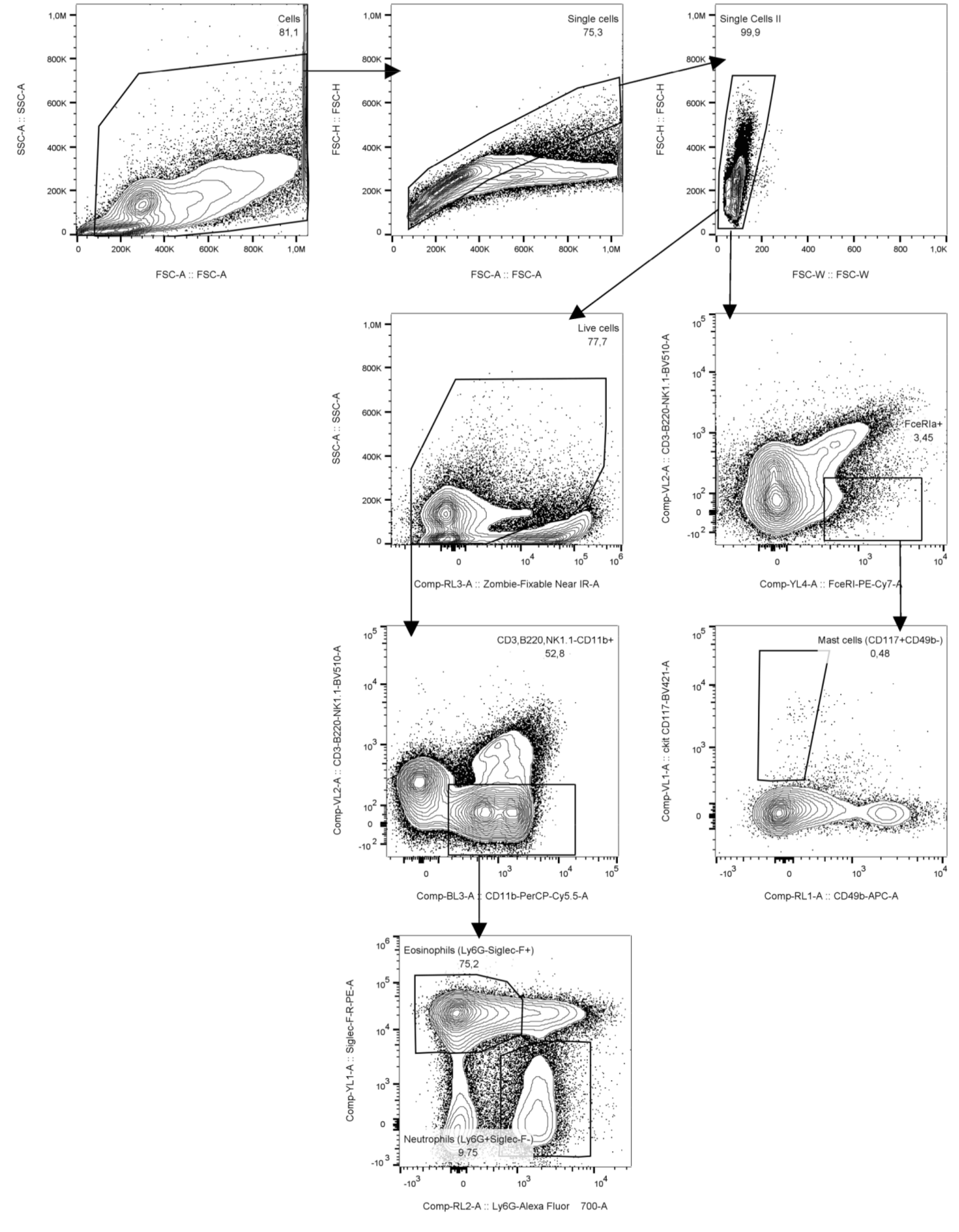

Figure S3. Flow cytometry gating strategy for the analysis of lung eosinophils, neutrophils and mast cells. Leukocytes from the lung were gated for singlets and live cells. Live cells were gated for CD3/NK1.1/B220 and CD11b. $CD3^-/NK1.1^-/B220^-/CD11b^+$ cells were gated for neutrophils ($CD3^-/NK1.1^-/B220^-/CD11b^+/Ly6G^+/Siglec\text{-}F^-$) and eosinophils ($CD3^-/NK1.1^-/B220^-/CD11b^+/Ly6G^-/Siglec\text{-}F^+$). Mast cells ($Fc\varepsilon RI\alpha^+/CD117^+/CD49^-$) were gated from singlets without prior dead cell exclusion. Gating is shown for a representative control mouse (PBS/alum only/OVA) **(a)** as well as a representative mouse

following the induction of AAI alone (PBS/OVA-alum/OVA) **(b)**. Gate frequencies relate to the respective parent gate.

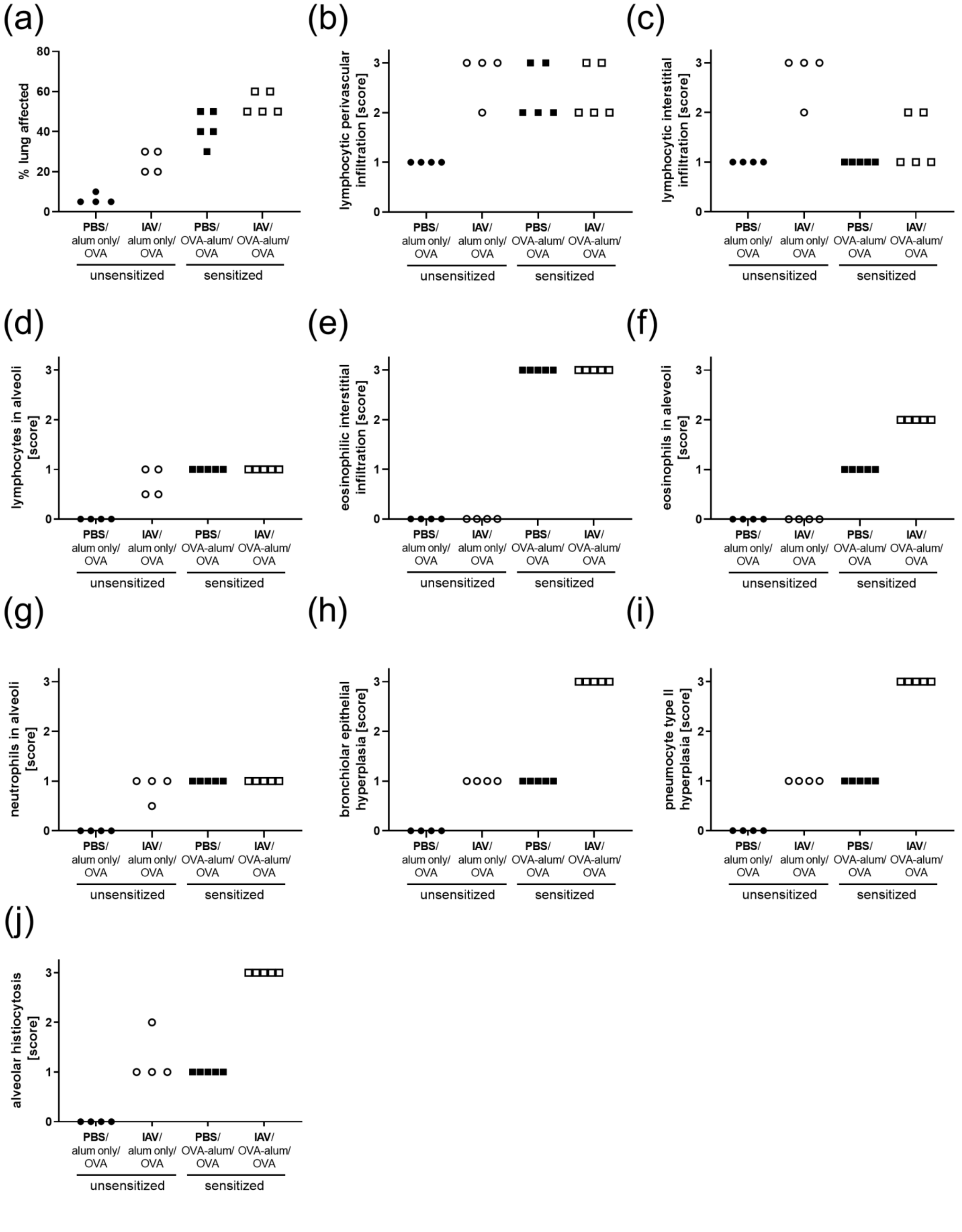

Figure S4. Histopathological analyses of lung tissue following resolution of IAV infection alone, AAI alone and AAI preceded by IAV infection. Mice were infected i.n. with a sublethal dose of IAV or treated with PBS on day 0, were sensitized against OVA i.p or mock-sensitized with alum only i.p. on days 14, 21 and 28 and challenged i.n. with OVA on days 35, 36 and 37. On day 39 post infection, lung

tissue sections were stained with hematoxylin and eosin and evaluated for the % lung affected **(a)**, lymphocytic perivascular infiltrations **(b)**, lymphocytic interstitial infiltrations **(c)** lymphocytes in the alveoli **(d)**, eosinophilic interstitial infiltrations **(e)**, eosinophils in the alveoli **(f)**, neutrophils in the alveoli **(g)**, bronchiolar epithelial hyperplasia **(h)**, pneumocyte type II hyperplasia **(i)** and alveolar histiocytosis **(j)**. Scores are shown for individual mice. 0 = not detected, 1 = mild, 2 = moderate, 3 = severe.

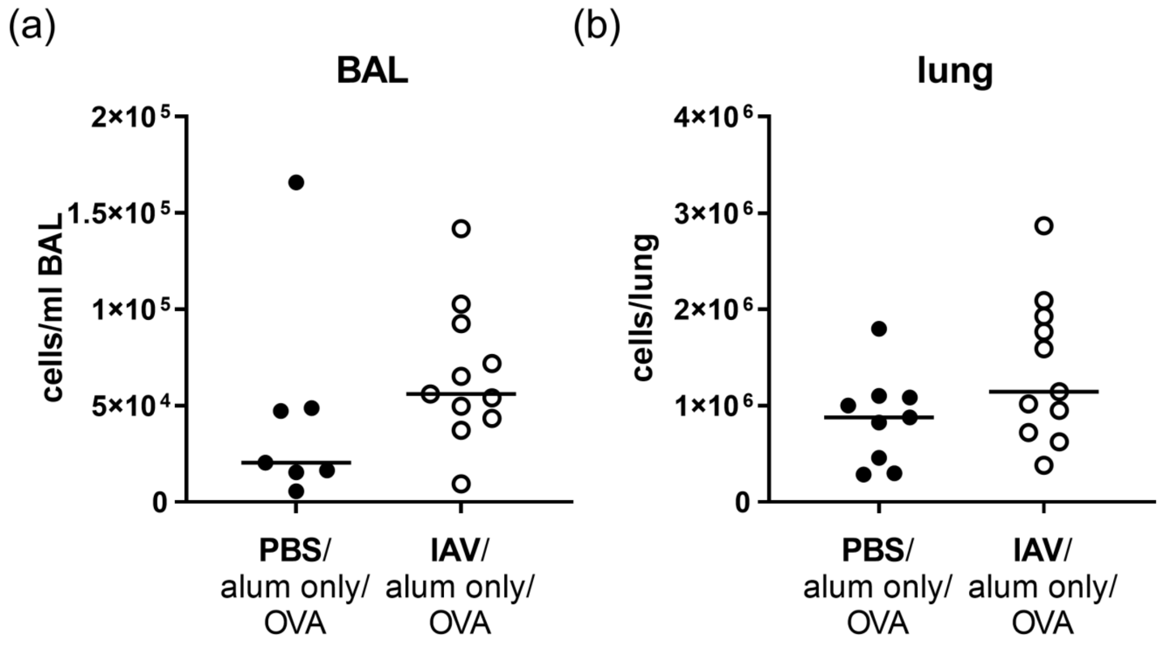

Figure S5. Resolved IAV infection does not significantly affect total cell numbers in the bronchoalveolar lavage (BAL) or the lung. Mice were infected i.n. with a sublethal dose of IAV or treated with PBS on day 0, were mock-sensitized (alum only i.p.; days 14, 21, 28) and treated i.n. with OVA (days 35, 36, 37). On day 39 post infection, total leukocyte numbers in the BAL (a) and lungs (b) were flow cytometrically determined. Data are shown for individual mice together with the group median. BAL data are compiled from at least two independent experiments and lung data are compiled from three independent experiments.

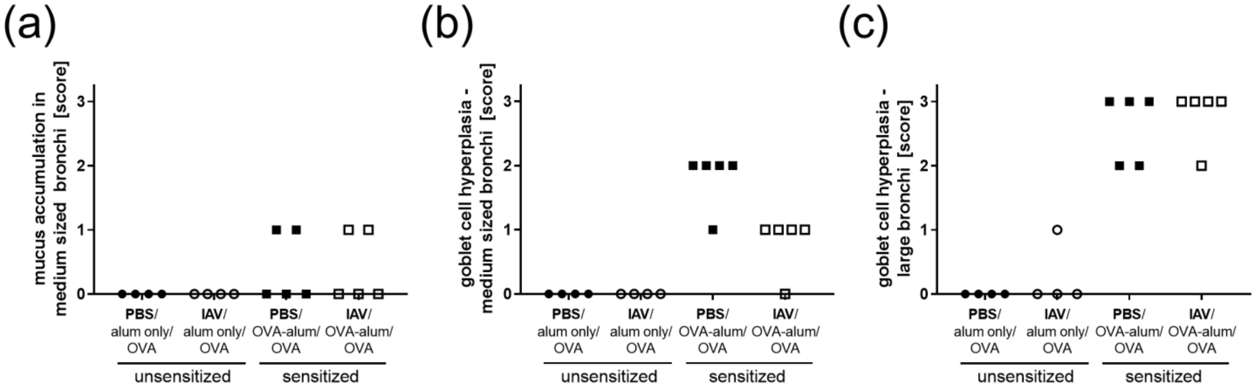

Figure S6. Histopathological analyses of mucus production following resolution of IAV infection alone, AAI alone and AAI preceded by IAV infection. Mice were infected i.n. with a sublethal dose of IAV or treated with PBS on day 0, were sensitized against OVA i.p or mock-sensitized with alum only i.p. on days 14, 21 and 28 and challenged i.n. with OVA on days 35, 36 and 37. On day 39 post infection, PAS (periodic acid-Schiff) reaction was performed on lung tissue sections and evaluated for mucus accumulation **(a)**, goblet cell hyperplasia in the medium sized bronchi **(b)** and goblet cell hyperplasia in the large bronchi **(c)**. Scores are shown for individual mice. 0 = not detected, 1 = mild, 2 = moderate, 3 = severe.

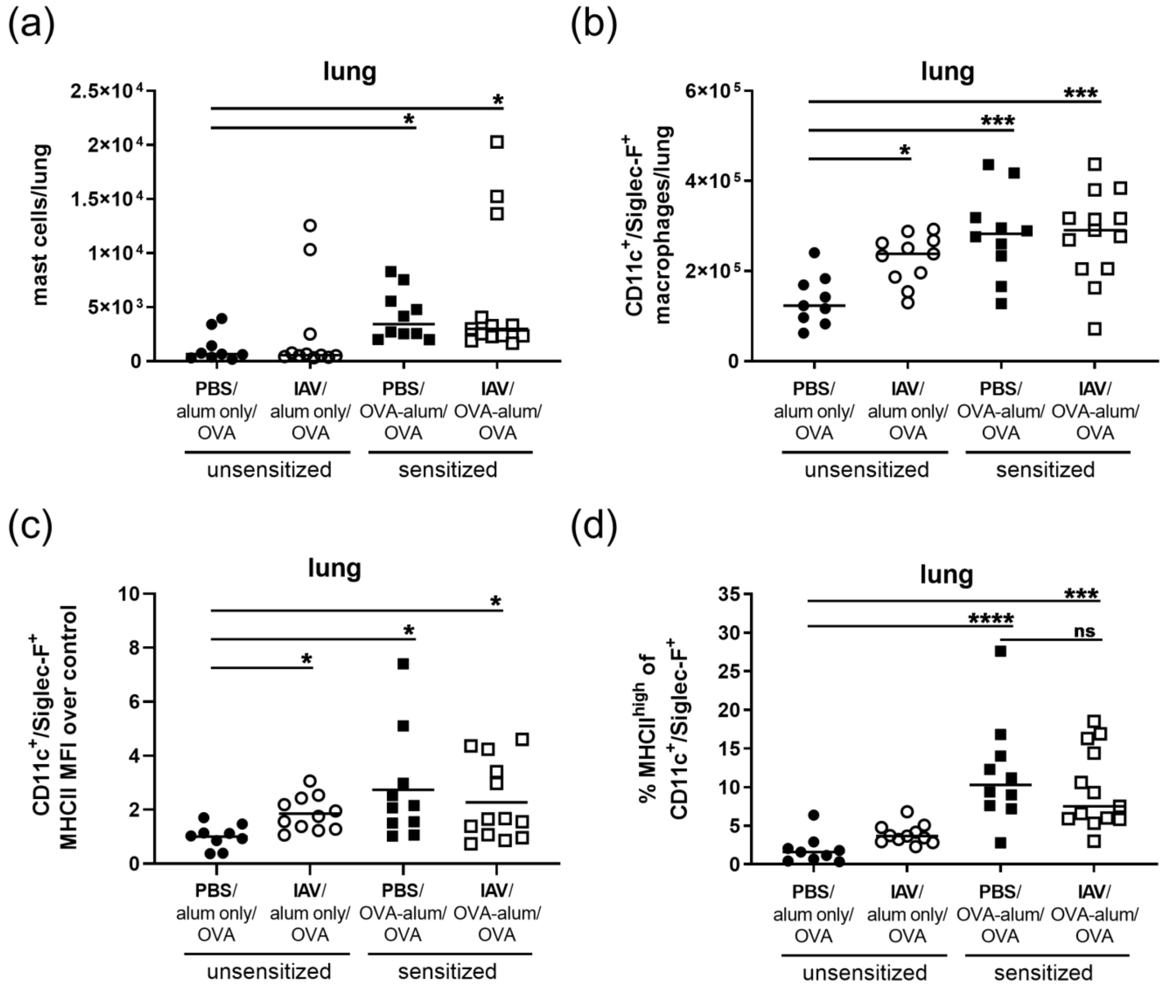

Figure S7. Resolved IAV infection does not affect lung mast cell numbers and macrophage numbers or macrophage MHCII expression in AAI. Mice were infected i.n. with a sublethal dose of IAV or treated with PBS on day 0, were sensitized against OVA i.p or mock-sensitized with alum only i.p. on days 14, 21 and 28 and all mice were challenged i.n. with OVA on days 35, 36 and 37. On day 39 post infection, mast cells (a) and CD11c+/Siglec-F+ macrophages (b) in the lungs as well as the MHCII expression (MFI, median fluorescence intensity) of the CD11c+/Siglec-F+ macrophages (c) and the frequency of MHCII^{high} CD11c+/Siglec-F+ macrophages in the CD11c+/Siglec-F+ macrophage population (d) were flow cytometrically analyzed. Data are shown for individual mice together with the group median and are compiled from three independent experiments. * $p < 0.05$, *** $p < 0.005$, ns = not significant.
